# Fahr’s syndrome: Evaluation of bilateral basal ganglia and calcifications in other brain regions: A report of 3 cases

**DOI:** 10.1016/j.radcr.2025.10.090

**Published:** 2025-11-29

**Authors:** Soheil Mirzaei, Zahra Motaghed, Sara Arab Baferani, Reza Naseri, Farzin Lotfi

**Affiliations:** a Department of Anatomy, Faculty of Medical Sciences, Tarbiat Modares University, Tehran, Iran; b Department of Radiology, Shahid Sattari Hospital, Shahid Beheshti University of Medical Sciences, Tehran, Iran; c Department of Emergency Medicine, Shahid Sattari Hospital, Shahid Beheshti University of Medical Sciences, Tehran, Iran; d Department of Radiology, Shohada-E Tajrish Hospital, Shahid Beheshti University of Medical Sciences, Tehran, Iran

**Keywords:** Fahr’s syndrome, Basal ganglia calcification, Neuropsychiatric disorders, Hypoparathyroidism, Case report

## Abstract

Fahr’s syndrome is a rare neurological disorder characterized by abnormal intracranial calcifications, most commonly affecting the basal ganglia. We report 3 cases—2 females aged 71 and 72, and 1 male aged 47—who were incidentally found to have bilateral basal ganglia calcifications on brain CT-scans. The first patient had a history of total thyroidectomy and parathyroidectomy; the second had prior psychiatric illness and was evaluated after a motor vehicle accident; the third was undergoing chemotherapy for hepatocellular carcinoma and presented with generalized weakness. Stroke was excluded in all cases, and each patient was discharged in stable condition. These cases highlight the importance of neuroimaging in identifying Fahr’s syndrome, especially in atypical presentations, and underscore the need for thorough metabolic and clinical evaluation to guide diagnosis and management.

## Introduction

Fahr’s syndrome is a rare neurological condition characterized by abnormal calcification in various brain regions, including the basal ganglia, thalamus, and hippocampus. The disorder may be inherited or sporadic, with an estimated prevalence of less than 1 per million individuals [[1], [2]].

It presents with a broad spectrum of neurological and psychiatric symptoms, including Parkinsonism, choreoathetosis, dystonia, motor incoordination, and dysarthria. Affected patients may also exhibit signs of depression, anxiety, visual and auditory hallucinations, delusions, mania, personality and behavioral disturbances, schizophrenia-like psychosis, and delirium [1]. Additionally, cognitive deficits are common and may impair verbal memory, spatial perception, executive planning, attention, concentration, and visuoconstructive skills [1,2].

Radiologic diagnosis of Fahr’s syndrome relies on detecting bilateral calcifications in the striatum, pallidum, and dentate nuclei. These findings are typically associated with progressive cognitive decline and movement disorders, without evidence of metabolic, infectious, toxic, or traumatic causes [3]. Recent studies have suggested a correlation between Fahr’s syndrome and metabolic or systemic disorders such as hypoparathyroidism [3,4]. Moreover, possible associations with Coronavirus Disease 2019 (COVID-19) have been highlighted in recent research [4]. Dental evaluations in affected individuals have revealed anomalies including delayed tooth eruption, enamel hypoplasia, and supernumerary teeth. These findings may serve as early clinical indicators of the syndrome [5]. In this report, we present 3 cases of patients with bilateral basal ganglia calcifications and discuss their clinical presentations, imaging findings, and diagnostic considerations related to Fahr’s syndrome.

## Case report 1

**Fig. 1:**
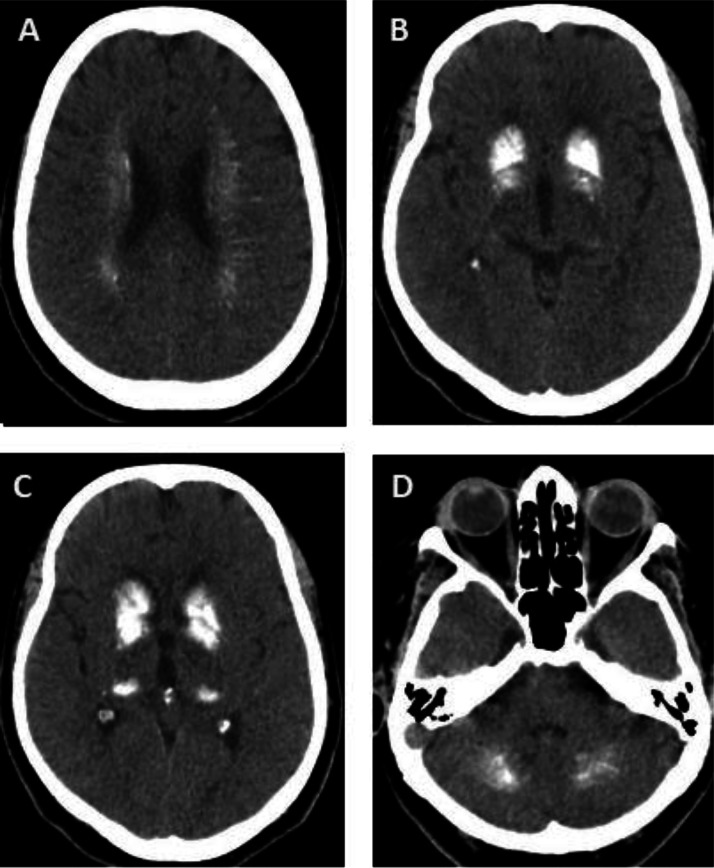
(A–D) Brain computed tomography (CT) images demonstrate extensive and bilateral calcifications in deep cerebral structures, including the corona radiata (A), basal ganglia (B and C), and dentate nuclei of the cerebellum (D). These calcifications are symmetrically distributed and predominantly affect regions involved in motor coordination and cognitive processing. The imaging findings suggest abnormal calcium deposition, which may be associated with underlying metabolic or neurological disorders and warrant thorough clinical evaluation.

A 72-year-old female patient was admitted to the hospital with complaints of generalized weakness. Initial clinical assessment revealed dysarthria and disorganized speech. For further evaluation, a brain computed tomography (CT) scan was performed, which showed bilateral calcification of the basal ganglia, as well as additional calcifications involving other areas, including the dentate nuclei of the cerebellum (Fig. 1). These radiological findings raised suspicion for Fahr’s syndrome. The patient was referred to a neurology center to rule out cerebrovascular accident (CVA). Following comprehensive assessment, CVA was excluded. She was subsequently discharged in stable and satisfactory condition.

Medical history review indicated that the patient had undergone total thyroidectomy and parathyroidectomy more than 40 years earlier due to a thyroid nodule. Given the surgical history and imaging results, further investigation was warranted to explore a possible link between metabolic alterations and intracranial calcifications.

## Case report 2

A 47-year-old male patient presented to the emergency department with complaints of generalized weakness and fatigue. He denied any history of neurological or psychiatric disorders. His medical records revealed a diagnosis of hepatocellular carcinoma, for which he was undergoing chemotherapy.

**Fig. 2:**
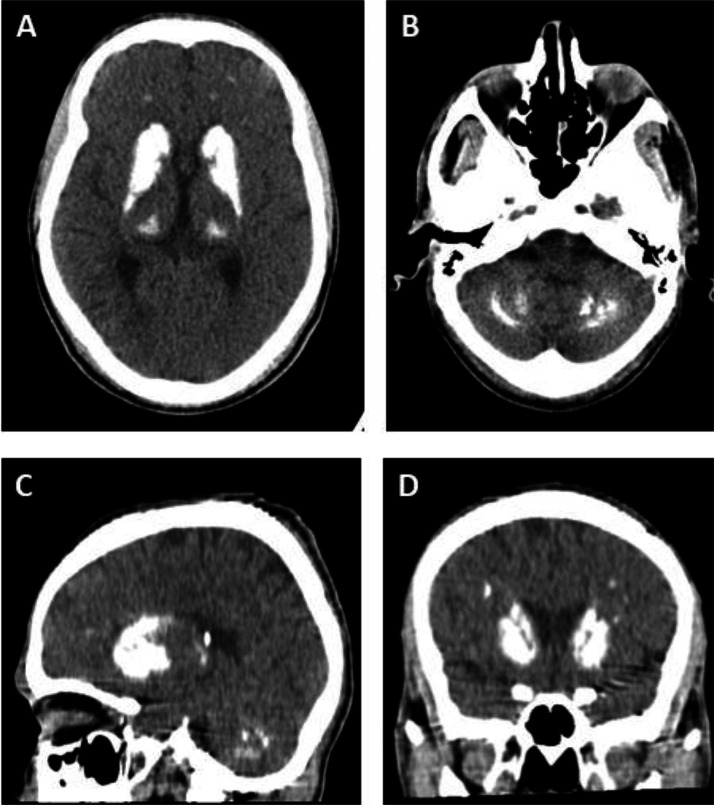
Brain computed tomography (CT) images reveal bilateral and symmetrical calcifications in key subcortical regions. (A) Axial CT scan showing bilateral calcifications in the basal ganglia. (B) Axial CT scan demonstrating symmetric calcifications in the dentate nuclei of the cerebellum. (C) Sagittal CT view highlighting calcified deep brain structures. (D) Coronal CT view confirming symmetrical calcium deposition across basal ganglia and cerebellar regions. These findings reflect abnormal calcium accumulation in areas essential for motor regulation and cerebellar coordination.

During the initial evaluation, a brain CT-scan was performed incidentally to rule out acute intracranial pathology. The imaging revealed symmetrical calcifications in the dentate nuclei of the cerebellum and bilateral basal ganglia (Fig. 2). These radiological findings were suggestive of Fahr’s syndrome, despite the absence of neuropsychiatric symptoms.

Following supportive care in the emergency department, the patient was discharged in stable condition.

## Case report 3

**Fig. 3:**
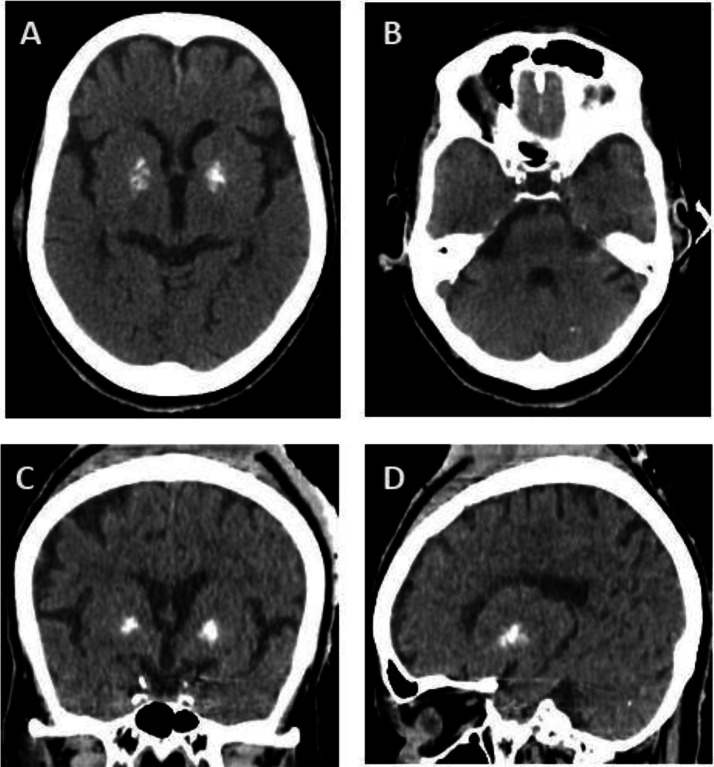
(A–D) Brain computed tomography (CT) scans reveal bilateral calcifications in the basal ganglia, accompanied by limited and scattered calcium deposits in the cerebellum. Compared to the first case, the extent of calcification is notably lower, with cerebellar involvement appearing as small and localized foci. Panels A and B represent axial views, panel C is a coronal view, and panel D illustrates the sagittal plane.

A 71-year-old female was brought to the emergency department via ambulance following a motor vehicle accident. Brain CT-scan imaging, performed incidentally, revealed bilateral calcification of the basal ganglia (Fig. 3).

Review of her medical history indicated episodes of psychological distress and aggression. She had a history of psychiatric medication use, and her daughter had also reported neurological concerns. The neuroimaging confirmed bilateral basal ganglia calcifications—a finding that necessitates further diagnostic workup to evaluate potential etiologies.

## Discussion

Fahr’s syndrome is a rare neurological disorder defined by symmetrical and bilateral calcifications in several brain regions, including the basal ganglia, thalamus, dentate nuclei, and cerebral cortex. The condition may result from metabolic imbalances, genetic mutations, or other unidentified etiologies. In some cases, it is secondary to disorders such as hypoparathyroidism [3].

Genetic studies have identified mutations in the SLC20A2 gene on chromosome 8 as the primary cause of familial cases. These are predominantly inherited in an autosomal dominant pattern, although autosomal recessive transmission and sporadic cases have also been reported [1].

Recent findings suggest that Fahr’s syndrome, in addition to its established genetic basis, may be associated with certain metabolic disorders—most notably hypoparathyroidism—and with neuropsychiatric conditions [6,7]. Early diagnosis and effective management require precise brain imaging, genetic analysis, and psychiatric evaluation, especially in patients presenting with psychotic features [7]. The broad range of clinical manifestations, from cognitive and motor disturbances to behavioral changes and anxiety, underscores the need to investigate its underlying pathophysiology [8].

During the COVID-19 pandemic, several studies reported that hypocalcemia may be linked to increased disease severity in Coronavirus Disease 2019 (COVID-19) [4]. Additionally, dental examinations in affected individuals have revealed atypical anomalies such as delayed tooth eruption and enamel hypoplasia [5]. These findings, in conjunction with genetic and metabolic evidence, emphasize the need for further research to better understand Fahr’s syndrome and to develop targeted therapeutic strategies [8].

CT-scan remains the most effective modality for detecting intracranial calcifications in patients with Fahr’s syndrome [3,[7], [8], [9]]. This imaging technique clearly visualizes calcium deposits within the basal ganglia, thalamus, and other affected brain regions, facilitating differential diagnosis from other neurological disorders [8]. Common sites of calcification include the globus pallidus, putamen, caudate nucleus, internal capsule, dentate nuclei of the cerebellum, thalamus, and cerebral white matter [2].

In addition to CT imaging, biochemical assessment of serum calcium, phosphate, parathyroid hormone, and vitamin D levels is essential to determine the underlying etiology—particularly in cases involving hypoparathyroidism [3,9].

Clinical studies have demonstrated a strong correlation between the severity of neurological symptoms and the extent of cerebral calcification. In advanced cases, patients may exhibit pronounced neuropsychiatric symptoms, personality changes, and features resembling schizophrenia [1,10]. Consequently, neuroimaging and screening in at-risk individuals is recommended prior to symptom onset [1]. Furthermore, certain endocrine abnormalities—such as thrombocytopenia—have been observed in some patients, increasing the risk of intracranial hemorrhage during seizure episodes [9].

More than 40% of patients with Fahr’s syndrome exhibit psychotic symptoms, including delusions, auditory and visual hallucinations, anxiety disorders, and obsessive-compulsive tendencies. Among these, psychotic manifestations are considered the most prevalent clinical presentation of the disease. These findings underscore the potential for misdiagnosis, particularly in younger individuals with no known family history, where Fahr’s syndrome may be mistaken for primary psychiatric disorders such as schizophrenia. This highlights the importance of brain imaging in patients presenting with atypical psychotic symptoms, as early identification of underlying calcifications can significantly alter clinical management [6].

Recent studies have shown that some patients with Fahr’s syndrome exhibit distinct dental anomalies, including delayed tooth eruption, enamel hypoplasia, supernumerary teeth, and root resorption. Genetic analysis of these individuals has implicated mutations in KIF1A, FZD8, and PDGFA genes as potential contributors to these abnormalities. In addition, a review of 22 scientific articles confirmed the association between Fahr’s syndrome and both hypoparathyroidism and pseudohypoparathyroidism, conditions that can significantly affect tooth development and root morphology. These findings underscore the importance of dental evaluation as a potential early clinical marker for the diagnosis of Fahr’s syndrome [5].

Multiple studies have demonstrated that Fahr’s syndrome may be associated with certain autoimmune and endocrine disorders. One such example is autoimmune polyendocrine syndrome type 1 (APS-1), which includes hypoparathyroidism, adrenal insufficiency, and mucocutaneous candidiasis. Although rare, this condition has been identified as a potential underlying contributor to Fahr’s syndrome. Additionally, a possible association with neuromyelitis optica spectrum disorder (NMO-SD) has been reported. Affected individuals may present with hemiparesis, dysarthria, and elevated aquaporin-4 (AQP-4) antibodies, leading to extensive transverse myelitis in the cervical spinal cord [11].

Other research confirms that secondary hypoparathyroidism following thyroid surgery may trigger Fahr’s syndrome. Damage to parathyroid tissue during surgery can result in hypocalcemia and hyperphosphatemia, ultimately facilitating abnormal calcium deposition in the brain [12].

During the COVID-19 pandemic, brain CT of a 63-year-old patient presenting with prolonged fever, dyspnea, and dry cough revealed widespread basal ganglia calcification. Laboratory results indicated severe hypocalcemia, hyperphosphatemia, and low parathyroid hormone levels, supporting a link between endocrine dysregulation and disease severity in Fahr’s syndrome patients [4].

Conversely, another study reported a 27-year-old woman with chronic headaches, seizures, and severe anxiety. Her serum calcium and phosphate levels, as well as endocrine function, were within normal limits. Genetic testing ruled out mutations in the SLC20A2 and PDGFB genes [8].

Collectively, these findings highlight the complexity of Fahr’s syndrome and reinforce the necessity of thoroughly investigating metabolic, genetic, and immunological factors for accurate diagnosis and effective management.

Although no definitive treatment exists for Fahr’s syndrome, current therapeutic approaches focus on symptomatic management. These include the use of anticonvulsants, antidepressants, calcium and vitamin D supplementation for endocrine regulation, and antipsychotic medications [5]. In cases presenting with psychotic symptoms, atypical antipsychotics such as paliperidone have been recommended [6]. For patients exhibiting dental anomalies, genetic testing and cone-beam computed tomography (CBCT) are suggested for further evaluation [5].

In individuals with parkinsonism-like symptoms, administration of antiparkinsonian and antiepileptic drugs has shown clinical benefit [6]. Among patients with hypoparathyroidism, combined treatment with calcium and vitamin D supplements, along with phosphate-lowering agents, may help alleviate neurological manifestations [7,13]. For example, an 11-year-old girl experiencing recurrent seizures, muscle weakness, and psychotic symptoms showed partial clinical improvement after receiving calcium supplementation and anticonvulsant therapy [13].

Further research is essential to elucidate the mechanisms underlying cerebral calcification and to improve therapeutic strategies for Fahr’s syndrome. These investigations may enable identification of contributing factors and support the development of targeted treatment approaches [13]. Early diagnosis—particularly in patients presenting with psychotic symptoms or motor disturbances—can prevent delays in intervention and enhance disease management outcomes [6].

Timely diagnosis and prevention of Fahr’s syndrome progression require monitoring calcium metabolism and performing brain imaging. This is especially important in young patients with unusual clinical features [3]. Genetic counseling is recommended for individuals at risk to enable effective screening and proactive management in familial cases [1].

In addition, emerging associations between Fahr’s syndrome and Coronavirus Disease 2019 (COVID-19), as well as dental anomalies, have raised new clinical questions that warrant broader investigation [4,5].

Advancements in neuroimaging techniques and genetic diagnostics continue to improve the accuracy of Fahr’s syndrome detection and inform personalized treatment options. This report describes 3 patients with bilateral basal ganglia calcifications. Their clinical features, imaging results, and diagnostic aspects of Fahr’s syndrome are discussed [13].

## Conclusion

Fahr’s syndrome is a rare neurological disorder characterized by extensive calcification in deep brain structures, with potential links to metabolic, genetic, and neuropsychiatric factors. Early detection through brain imaging and evaluation of calcium metabolism plays a critical role in disease management. Future research is essential to improve understanding of disease mechanisms and to develop targeted therapeutic strategies.

## Patient consent

Written informed consent was obtained from the patient for publication of this case report and accompanying images. A copy of the written consent is available for review by the Editor-in-Chief of this journal on request.
